# Role of Endothelial Glucocorticoid Receptor in the Pathogenesis of Kidney Diseases

**DOI:** 10.3390/ijms222413295

**Published:** 2021-12-10

**Authors:** Jarosław Przybyciński, Sylwester Drożdżal, Leszek Domański, Violetta Dziedziejko, Andrzej Pawlik

**Affiliations:** 1 Department of Nephrology, Transplantology and Internal Medicine, Pomeranian Medical University in Szczecin, Powstańców Wlkp. 72, 70-111 Szczecin, Poland; jarpe85@gmail.com (J.P.); starkdrozd@wp.pl (S.D.); domanle@pum.edu.pl (L.D.); 2 Department of Biochemistry and Medical Chemistry, Pomeranian Medical University in Szczecin, Powstańców Wlkp. 72, 70-111 Szczecin, Poland; viola@pum.edu.pl; 3 Department of Physiology, Pomeranian Medical University in Szczecin, Powstańców Wlkp. 72, 70-111 Szczecin, Poland

**Keywords:** glucocorticoids, glucocorticoid receptor, kidney, endothelium, diabetic kidney disease, glomerulopathy

## Abstract

Glucocorticoids, as multifunctional hormones, are widely used in the treatment of various diseases including nephrological disorders. They are known to affect immunological cells, effectively treating many autoimmune and inflammatory processes. Furthermore, there is a growing body of evidence demonstrating the potent role of glucocorticoids in non-immune cells such as podocytes. Moreover, novel data show additional pathways and processes affected by glucocorticoids, such as the Wnt pathway or autophagy. The endothelium is currently considered as a key organ in the regulation of numerous kidney functions such as glomerular filtration, vascular tone and the regulation of inflammation and coagulation. In this review, we analyse the literature concerning the effects of endothelial glucocorticoid receptor signalling on kidney function in health and disease, with special focus on hypertension, diabetic kidney disease, glomerulopathies and chronic kidney disease. Recent studies demonstrate the potential role of endothelial GR in the prevention of fibrosis of kidney tissue and cell metabolism through Wnt pathways, which could have a protective effect against disease progression. Another important aspect covered in this review is blood pressure regulation though GR and eNOS. We also briefly cover potential therapies that might affect the endothelial glucocorticoid receptor and its possible clinical implications, with special interest in selective or local GR stimulation and potential mitigation of GC treatment side effects.

## 1. Introduction

Glucocorticoids (GCs) are multifunctional hormones affecting the human metabolism, the immunological system, reproduction, circadian rhythm and several other vital functions. This implies the importance of GCs in the treatment of a wide spectrum of diseases such as autoimmune and inflammatory processes and malignancies [1,2]. For many years, they have been a staple in the treatment of a variety of kidney diseases such as glomerulonephritis [3]. Despite this success, they can cause several side effects such as infections or compromised metabolism [4]. In some cases, treatment resistance might occur [4]. Those factors increase interest in GC function and signalling in different cells and tissues to minimise side effects and increase the efficacy of GC curation. On the other hand, novel studies have unravelled the roles of GCs and their receptors in the pathology of different diseases and processes [4]. Furthermore, GCs play a variety of roles, sometimes even opposing ones, in different tissues and organs [5]. Research in recent years brought evidence of multifactorial effect of sex hormones and mineralocorticoids on kidney function and structure, especially in diabetic kidney disease [6,7]. Moreover, other studies revealed a wide-array of functions of nuclear receptors in podocytes [8]. One novel study drew attention to the endothelial glucocorticoid receptor function in diabetic kidney disease [9]. In this review, we will focus on the role of the endothelial glucocorticoid receptor (GR) in the pathogenesis of kidney diseases (Table 1).

In humans, GCs are produced mainly by the adrenal glands under the regulation of the hypothalamic–pituitary–adrenal axis and their secretion depends on various factors such as stress or daily rhythm [2]. There is also evidence of local production of GCs in other organs, mainly immunocompetent organs such as the skin, thymus or intestines, although their function is still uncertain [10]. Studies have shown that rat kidney tissue can produce steroid hormones, but this process is unknown in humans [11].

GCs are transported in the blood in inactive forms bound to GC-binding globulin and albumin. After detachment from binding proteins, GCs can penetrate cell membranes and bind with GC receptors in the cytoplasm. Before attaching to receptors, GC function can be regulated by the enzymatic reaction of dehydrogenases: 11β-hydroxysteroid dehydrogenase 1 and 2 in humans causing, respectively, transformation to active cortisol or inactive cortisone. Those enzymes play important, site-specific roles in GC function, with 11β-hydroxysteroid dehydrogenase 1 dominant in the vascular endothelium [4,12].

The GR consists of three functional domains: an N-terminal transactivation domain, a central DNA binding domain and a C-terminal ligand-binding domain. Several alternative isoforms—GRα, GRβ, GRγ, GR-A and GR-P—can be formed through alternative splicing. While GRα is considered as the main active form, other variants either inhibit GR signalling or fail to bind GCs and might be responsible for GC resistance. The inactive form of GRα is located mainly in the cytoplasm and binds to several chaperones such as hsp90, hsp72, hsp 53 and immunophilins. After interaction with the ligand, it is translocated to the nucleus and can interact with DNA in dimeric or monomeric form. The GR can either promote transcription of genes—transactivation—or suppress it, causing transrepression, in several ways—independently or by interaction with other transcription factors and cofactors. GCs can also exert an almost immediate effect in several cells through a membrane-located GR. This pathway is related more to cell signalling rather than gene transcription and explains the rapid effect of GCs on the circulatory system or respiratory tract observed in clinic. Another important aspect of GR function is post-translational modifications which might partly describe the unique GC function in different cells [13,14].

A factor causing GR nuclear translocation and further genetic effects is shear stress. It is unique to endothelial cells and associated with cellular structure, especially nuclear lamina mechanical stimulus transduction, as has been shown in cell studies. It is postulated that unidirectional, non-turbulent arterial flow might exert an anti-inflammatory and anti-atherogenic effect through GR stimulation, independent of traditional receptor–ligand interaction [15,16]. A study has demonstrated GR stimulation by high doses of nitric oxide (NO) in bovine aortic endothelial cells. The authors suggested that stimulatory concentrations of NO can be achieved in an inflammatory milieu, revealing potential complicated feedback between NO and GCs in endothelial cells [17].

Kidney endothelium is responsible for a variety of processes vital for kidney health and function. Among them are angiogenesis, control of inflammation and leucocyte trafficking, regulation of vascular tone, haemostasis and coagulation. Endothelial cells (ECs) also control vascular smooth muscle cell proliferation and vascular permeability. ECs can easily change their phenotype to a pro-inflammatory and pro-thrombotic phenotype which plays an important role in the pathophysiology of kidney disease. Kidney ECs are a diversified population with different characteristics and function. We can highlight the glomerular endothelium, the microvascular endothelium of peritubular capillaries and the endothelium of larger vessels. Uniquely, the glomerular endothelium is fenestrated and covered with a thick, negatively charged glycocalyx and net of glycosaminoglycans [18]. ECs also have important metabolic features where glycolysis and fatty acid oxidation are not solely energy sources but possibly modulate the programming of different cell functions. Disturbance of either of the metabolic pathways can cause negative effects on such actions as angiogenesis and cell survival, or lead to fibrosis. Non-homogenic metabolism of various EC populations might partly explain organ-specific diabetic complications [19].

Animal studies show a distribution pattern of GR and mineralocorticoid receptor (MR) in the kidney. GR is located mainly in the glomeruli, proximal tubule and thick ascending limb, while MR is found in the cortical collecting duct and outer and inner medullary ducts [20,21]. Examination of human glomeruli showed the presence of GR in all cell types: endothelium, podocytes, mesangial cells and ECs [22].

The anti-inflammatory effect of GCs in endothelium can be achieved not only by transrepression of inflammatory genes such as NF-κB or AP-1, but also by cytoplasmic action. A study in HUVECs has demonstrated that GR stimulation activates MPK-1 phosphatase within 5 min, thus inhibiting the MAPK pathway and E-selectin expression [23,24]. Novel studies found another pro-inflammatory pathway inhibited by GR stimulation. Genomic studies of mouse lung ECs revealed the repression of several genes associated with Wnt signalling [25]. Moreover, GCs reduce expression of adhesion molecules (ICAM, VCAM and E-Selectin) on endothelial cells [26]. On the other hand, GCs can induce some pro-inflammatory genes such as TLR 2 and 4, and NLRP3, or reinforce STAT3 signalling [24].

**Figure 1:**
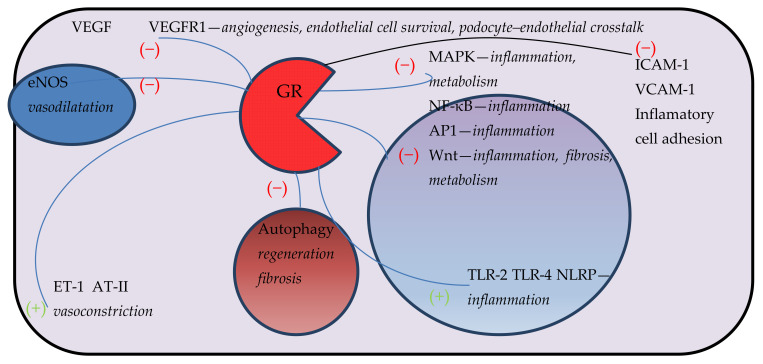
Selected effects of GR signalling in endothelial cells and its implication in kidney health and disease. Effect of GR signalling on various aspects of endothelial cell function showing inhibition of proinflammatory MAPK, NF-κB, AP1 and Wnt pathways as well as effect on vascular tone and VEGF-VEGFR1. “(−)” representing inhibition and (+) stimulatory effect. GCs lead transrepression of inflammatory genes such as NF-κB or AP-1 in endothelium. GR stimulation activates MPK-1 phosphatase and inhibits MAPK pathway and E-selectin expression. GCs can induce TLR 2 and 4, and NLRP3 genes. GCs can reduce VEGF and VEGF receptor 1 (VEGFR1) protein expression through the mTORC1 pathway. GR stimulation is associated with inhibition of the Wnt/β-catenin pathway. GCs can increase the secretion of ET-1 and AT-2 from ECs. GCs directly reduce eNOS transcription through GATA interaction. GCs reduce expression of adhesion molecules on endothelial cells. VEGF—vascular endothelial growth factor, VEGFR-1—vascular endothelial growth factor receptor 1, eNOS—endothelial nitric oxide synthase, ET-1—endothelin 1, AT-II—angiotensin II, MAPK—mitogen activated protein kinases, NF-κB—nuclear factor kappa B, AP1—activator protein 1, Wnt—Wnt signalling pathway, TLR—Toll-like receptor, NLRP—nuclear binding oligomerization domain, GR—glucocorticoid receptor, ICAM-1 intracellular adhesion molecule 1, VCAM-1—vascular cell adhesion 1.

GR stimulations exert an anti-angiogenic role in EC cultures. It is achieved by cytoskeletal alteration and inhibition of the formation of intracellular connections rather than influencing cell proliferation and migration. Furthermore, GC treatment does not change the VEGF mRNA level [27]. In a different study, HUVECs and bovine aortic ECs showed an attenuated response to VEGF and cell migration after GC treatment. It was related to upregulation of caveolin-1—a key protein associated with the function of plasmalemmal caveolae and response to various external stimuli [28]. HUVECs exposed to synthetic GCs show reduced VEGF and VEGF receptor 1 (VEGFR1) protein expression but increased sVEGFR2 synthesis through the mTORC1 pathway [29]. The latest studies on mice and murine cell lines have demonstrated that the anti-angiogenic effect of GR stimulation is also associated with inhibition of the Wnt/β-catenin pathway. Beyond that, endothelial GR knockout increases cell autophagy which, in turn, further stimulates Wnt signalling [30]. This demonstrates the multifaceted role of GR in endothelial cells (Figure 1).

## 2. Role of Endothelial Glucocorticoid Receptor in the Pathogenesis of Arterial Hypertension and Vascular Diseases

Hypertension is a common feature of Cushing syndrome which also affects nearly 20% of patients treated with GCs. The pathogenesis of this event is complex and still uncertain, affecting GC signalling in different tissues. Traditionally, it has been associated with mineralocorticoid function and sodium conservation, taking place mainly in the distal nephron, but novel data suggest that many other mechanisms might be involved in this process. Numerous studies, chiefly in animals, have pointed out the important role of vascular contractility affected by both endothelium and smooth muscle cells. In smooth muscles, GCs increase AT1 receptor concentration and change the flow of Na^+^ and Ca^2+^. In the central nervous system, GCs disturb neuronal NO release [31]. Salt-sensitive hypertension occurring in individuals with reduced kidney mass, both congenital and for example after unilateral nephrectomy, might be associated more with the function of GR than MR. A controlled study on rats fed a high-salt diet after unilateral nephrectomy showed that hypertension can be reversed by an MR antagonist but not an aldosterone synthase inhibitor. A significant disbalance of 11β-hydroxysteroid dehydrogenases, favouring the production of active GR in kidney tissue, has also been found. This can be explained by the fact that GCs have similar affinity for both GR and MR and their action in kidney is controlled by 11β-hydroxysteroid dehydrogenase 2 and local inactivation [32]. In so-called aldosterone-sensitive distal nephron, this enzymatic deactivation protects kidney epithelial cells from GC-mediated activation and allows for more precise regulation by mineralocorticoids [14]. A study on MR knockout mice demonstrated that expression of the epithelial Na^+^ channel and improvement of mineral balance can be mediated solely by GC therapy [33]. Interestingly, research on distal nephron GR knockout mice demonstrated that its function is not required for the development and maintenance of GC-induced hypertension [34]. Furthermore, another study on mice with local, tubular GR knockout showed only a transient effect on sodium handling despite the reduction in the sodium chloride cotransporter (NCC) expression [35]. On the other hand, GCs downregulate and inhibit vasopressin receptor, V2R, in the rat inner medullary collecting duct, causing water and sodium secretion after water overload [36].

GCs can cause various effects in ECs associated with blood pressure regulation. Primarily, they can increase the secretion of ET-1 and AT-2 from ECs, causing vascular smooth muscle contraction [26]. Secondarily, they affect NO action in many stages. GCs directly reduce eNOS transcription through GATA interaction in human cell lines. Furthermore, they increase eNOS mRNA degradation and reduce protein stability. In addition, GCs can lower intracellular Ca^2+^ mobilisation and tamper with the reaction of cells to such stimulants as ATP. The next potential mechanism reducing NO synthesis could be the inhibition of GTP cyclohydrolase—the enzyme responsible for the production of tetrahydrobiopterin, an eNOS cofactor. In animals and in cell cultures, GCs reduce prostacyclin production via phospholipase A2 and potentially COX-1 inhibition (in foetal cells) [37]. A study on mice demonstrated that endothelial GR knockout animals do not develop hypertension after dexamethasone administration. Although those mice had a higher baseline blood pressure (BP) than matched controls, their BP did not change significantly on administration of early or chronic oral dexamethasone. Endothelial GR knockout animals did not present elevated natriuresis in contrast to the control group. Both groups showed a similar decrease in NO production after dexamethasone treatment, but GR knockout mice had a decreased arteriole contractile response to the drug. Interestingly, vascular reactivity to phenylephrine was similar in both groups. In the case of circadian BP rhythm, while it changed significantly in both groups, leading to higher BP in resting hours, it partly returned to normal after 2–7 days in endothelial GR knockout mice [38]. A different study demonstrated that dexamethasone administration increases ROS production in HUVECs as another factor interfering with NO production [39].

In an animal sepsis model, endothelial GR plays a protective role by mitigating iNOS and eNOS activation and might be seen as a negative regulator of eNOS. Endothelial GR knockout mice had a higher mortality rate after LPS injection due to cardiovascular shock. Moreover, studies on HUVECs have shown that GR knockdown by siRNA augments the inflammatory response by upregulation of the NF-κB pathway [40]. This pathological effect could not be reversed by previous dexamethasone administration. In fact, endothelial GR knockout mice had worse prognosis with additional dexamethasone treatment. These studies indicate the importance of endothelial GR in the pathogenesis of septic shock and BP regulation in animal models [41]. On the other hand, previous studies have demonstrated that dexamethasone can activate eNOS in a non-genomic manner in a mouse model of ischaemia–reperfusion injury. In this model, GCs showed a protective role in reducing vascular inflammation and reducing the myocardial infarct area [42].

At the moment, it is assumed that atherosclerosis originates due to two events: EC damage and vascular remodelling. The consequences are increased endothelial permeability and recruitment of circulating inflammatory cells through ICAM-1 and VCAM-1 expression. This allows the migration of LDL into the vascular wall and further inflammation. Traditionally, an excess of GCs, as observed in Cushing syndrome, is connected with higher cardiovascular risk and development of atherosclerosis but novel studies of both animal models and cell lines bring conflicting results. Despite the known role of GCs in the downregulation of EC adhesion molecules and inhibition of IL-6, IL-8 and CCL which should be beneficial, some animal models show pro-atherogenic effects [26].

Endothelial GR is important in the pathophysiology of atherosclerosis. Studies using endothelial GR knockout mice fed with a high-fat diet showed greater atherosclerosis in comparison to wild genotype mice. It is postulated that the action of endothelial GR attenuates atherosclerosis and vascular inflammation [43]. Due to the multifactorial pathophysiology of atherosclerosis, the effect might be dependent on the different cells involved and other aspects [26].

## 3. Role of Endothelial Glucocorticoid Receptor in the Pathogenesis of Glomerulopathies

**Figure 2:**
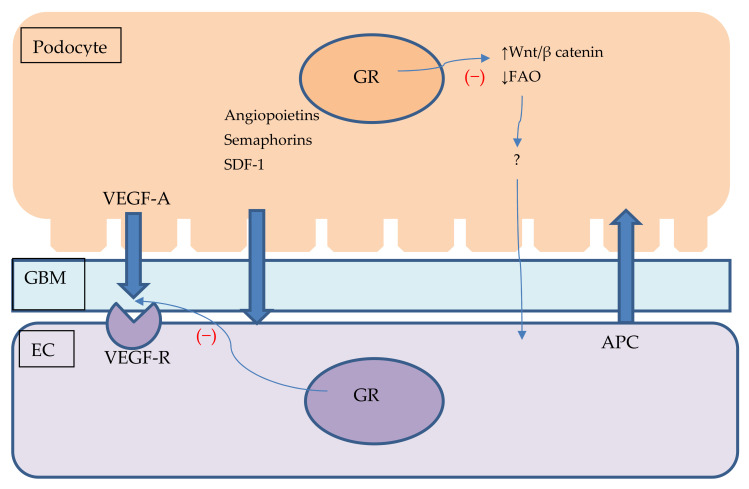
Podocyte–endothelial crosstalk and its potential connection with GR signalling. Figure shows podocyte and endothelial cell with glomerular base membrane between them. Podocytes produce VEGF, angiopoietins, semaphorins and SDF-1 which are secreted and influence endothelial cells homeostasis. ECs might secrete APC which protects podocytes against apoptosis. GR stimulation might impair VEGF signalling and influence podocyte Wnt/β catenin pathway and promote FAO. Lack of GR stimulation in podocytes might damage ECs through unknown mediator. APC—activated protein C, EC—endothelial cell, FAO—fatty acid oxidation, GBM—glomerular base membrane, SDF-1—Stromal cell-derived factor 1 VEGF—vascular endothelial growth factor, VEGF-R—vascular endothelial growth factor receptor, Wnt—Wnt signalling pathway.

For many years, GCs have been used as a cornerstone in the therapy of most types of glomerulonephritis, both primary and secondary, and in some, such as minimal change disease, they can be successfully used in monotherapy. In others, such as crescentic glomerulonephritis in vasculitis or severe cases of lupus nephritis, they fail to establish sustainable remission without the addition of other immunosuppressive drugs [3]. These heterogeneous clinical results have generated interest in the precise action of GCs in glomeruli. The therapeutic effect of GCs had been traditionally attributed to the mitigation of inflammation directly in immune cells. Novel studies unravel a more complex relationship. For example, GCs protect podocytes through stabilisation of the slit diaphragm and cytoskeleton and preserve podocyte differentiation after injury [44]. Moreover, mice with GR knockout in podocytes are more vulnerable to kidney injury by various factors and show worse proteinuria and podocyte foot process effacement. This has been attributed to defective cytoskeleton formation and migration after podocyte injury [45]. Interestingly, some studies have revealed a potential negative effect of GR stimulation. One study analysed GR inactivation (selective GR knockout or GR antagonist treatment) in kidney epithelial cells in a mouse model of crescentic glomerulonephritis. It led to a beneficial effect, reduced albuminuria and reduced crescent formation parallel to GC treatment results, but without any major side effects. This was achieved chiefly by inhibition of peripheral epithelial cell proliferation and activating a process associated with crescent formation [46]. The role of endothelial GR in glomerulopathies is less elucidated (Table 2). We already know that normal structure and function of endothelium is crucial for glomerular filtration and protection against thrombosis and inflammation. Based on the data from animals, cell lines and human tissue samples, we know that NF-κB signalling in ECs mediated by ANCA-stimulated neutrophils is a key pathogenetic factor causing ANCA-associated vasculitis [47]. Another important process responsible for appropriate glomerular filtration barrier function is podocyte–endothelium crosstalk (Figure 2). One of the major factors involved in this action is VEGF [48]. Mice overexpressing VEGFR in podocytes develop a nephrotic syndrome similar to minimal change disease observed in humans. It is mediated chiefly by podocyte foot process effacement without significant endothelial damage [49]. It has been previously demonstrated that plasma and urinary VEGF levels are elevated during the onset of nephrotic syndrome in humans and decline after GC treatment [50]. On the other hand, VEGF depletion can cause endothelial injury and proteinuria in a process called endotheliosis. It is assumed as a main pathophysiological lesion in pre-eclampsia or anti-VEGF cancer treatment complications [48,51,52].

Glomerular ECs compared with other ECs produce more fibrinolytic factors and have a different metabolic profile, leading to their vulnerability to oxidative stress and membrane lipid peroxidation [53]. Damage to ECs is primarily responsible for the pathogenesis of lupus nephritis, vasculitis, TMA and antibody-mediated rejection of kidney transplant [18,54,55].

Previous studies have demonstrated a potent anti-apoptotic effect of GCs in cultured bovine ECs. Exposure of cells to TNF-α or LPS causes apoptosis and this effect is inhibited by the addition of dexamethasone. There is a time window up to 18 h after cell insult when dexamethasone application shows a protective outcome. The nature of this reaction is still unclear. It has been clearly attributed to GR action and processes upstream of caspase activation, mainly changes in the composition of bcl-2-related proteins. On the other hand, the anti-apoptotic effect could be only partly linked with the early stages of apoptosis activation and mitochondrial permeability transition. Similar results of an anti-apoptotic effect of dexamethasone have been observed in an EC line exposed to fluvastatin [56,57,58]. 

Some types of glomerulonephritis, for example, those associated with vasculitis, cannot be treated effectively with GC monotherapy. An insight into this phenomenon has been given by analysing the effect of GCs in different cells. A study on cell cultures comparing the reaction to GCs in monocytes and ECs in standard and inflammatory milieus showed a contrasting result. In monocytes, GCs suppressed pro-inflammatory genes such as TNFSF10, IL1B and CCL5 and induced anti-inflammatory genes such as IL1R2, DUSP1, FPR1 and FKBP5. Interestingly, GCs failed to present this effect in HUVEC cultures. Further analysis and gene profiling revealed that while GCs suppressed 22% to 28% of immune response-associated genes in monocytes, they were able to suppress only 2% of those genes in HUVECs. This difference in GC action could not be explained by different expression of GR or its impaired translocation but might be associated with decreased induction of SAP30—a subunit of the Sin3A–HDAC corepressor complex [59]. Under other conditions, previous studies have shown that GCs can decrease activation of the MAPK signalling pathway, providing an anti-inflammatory effect in human microvascular ECs acquired from lungs. These results have not been tested in ECs acquired from kidneys [60]. In a different study, GCs inhibited IL-6 production but failed to interact with VCAM-1 induction in HUVECs under inflammatory conditions. This could be explained by the different coactivators necessary for GR–NF-κB interaction with IL-6 and VCAM-1 promotors [61].

It is also important to note that other nuclear receptors such as vitamin D receptor, retinoic acid receptor α and oestrogen receptors play important role in podocyte function and they interaction with GR in case of podocyte injury and homoeostasis might have further clinical implications [8,62].

## 4. Role of Endothelial Glucocorticoid Receptor in the Pathogenesis of Diabetic Kidney Disease

Although diabetic kidney disease is a main cause of end-stage renal disease in most developed countries, its aetiology is multifactorial and not fully understood [63]. As an example, only about 30% of diabetic patients will develop nephropathy, which suggests the existence of some unknown genetic or environmental protective factors. While diagnosis is often based on clinical symptoms and laboratory findings the most accurate way to diagnose and stage the disease is kidney biopsy [64,65]. Besides mesangial matrix expansion, glomerular sclerosis and podocyte injury, the vascular system is one of the key structures affected by the disease. Notably, early stages of diabetic kidney disease are associated with neoangiogenesis; late stages with vascular rarefaction and fibrosis [66]. One of the early signs of diabetic kidney disease is albuminuria. Although generally asymptomatic, it increases severe complications and risk of death. It is considered a symptom of endothelium injury. Diabetic kidney disease is strongly associated with endothelial damage. Hyperglycaemia induces the apoptosis of ECs. Other typical EC lesions occurring in diabetes are reduced fenestration, glycocalyx disruption and reduced NO availability [18]. On the other hand, diabetes affects a plethora of kidney cells and in the case of glomerular damage, the injury involves podocytes, ECs and mesangial cells. Novel studies show the rising importance of podocyte–endothelial crosstalk in homeostasis of the glomeruli. Those cells interact with each other through a variety of paracrine substances. Podocytes produce VEGF-A which binds with endothelial VEGF receptor 2 (VEGFR2) [67]. VEGF is mainly secreted by podocytes and stimulates ECs in a paracrine route but seems seem dispensable to podocytes by themselves [67]. There is a rising body of evidence showing the importance of VEGF–VEGFR2 balance in the glomeruli as well as different, diabetic milieus affecting this system. Different teams of researchers have demonstrated that both overexpression and depletion of VEGF signalling can lead to pathological proteinuria and glomerular injury. Another study showed that VEGF isoform overexpression in eNOS knockout mice can cause glomerular sclerosis, glomerular basement membrane thickening and vascular damage such as that observed in advanced diabetic kidney disease [68,69,70,71]. Moreover, diabetic kidney disease in mice has been linked with impaired eNOS synthesis and can be improved by supplementation with NO precursors [72,73]. All this shows the importance of interplay between glomerular cells and signalling pathways in the pathology of diabetic kidney disease. While there are no direct studies regarding the role of endothelial GR signalling in this relationship, we already know that GCs can affect both eNOS and VEGF systems. Other factors affecting ECs produced by podocytes are angiopoietins, semaphorins and SDF-1. ECs can inhibit podocyte apoptosis and inflammation by secreting activated protein C. Their role is so far inadequately understood [74]. Novel studies shed more light on the role of GR in diabetic kidney disease. Recently, there has been increasing interest in the role of Wnt signalling in the kidney. The canonical part of the Wnt/β-catenin pathway is generally regarded as profibrotic and involved in the pathogenesis of chronic kidney diseases, while the non-canonical part might be associated with inflammation and atherosclerosis [25,75]. It is also important in podocyte homeostasis. Studies of animal diabetic models have shown the complicated role of the Wnt/β-catenin pathway. While its activation protects podocytes from apoptosis, it also promotes podocyte detachment and loss [76]. A recent study of diabetic mice in which the GR gene of isolated podocytes was knocked out showed greater fibrosis of their kidneys. It has also been associated with increased Wnt/β-catenin signalling but also impaired fatty acid oxidation (FAO). Interestingly, there is also greater endothelial damage in the form of endothelial to mesenchymal transition not only in kidney samples but also isolated ECs treated with conditioned media from GR knockout podocytes. This once again shows the importance of podocyte–endothelium crosstalk, although direct mediators of it are yet to be found [9]. We already know that the Wnt/β-catenin pathway is induced by complement factor fragments in glomerular ECs and can lead to endothelial to mesenchymal transition in a rat diabetes model [77]. Endothelial to mesenchymal transition is regarded as one of the main sources of fibroblasts, contributing to renal fibrosis and end-stage kidney disease. A recent study found that impaired GR function in ECs might be one of the reasons for these phenotype shifts. GR knockout diabetic mice had greater fibrosis of renal tissue and increased markers of EndMT. Those animals also had induced canonical Wnt signalling and adding Wnt inhibiting particles improved fibrosis. Wnt overstimulation has been associated with the induction of Snail1 and HIF1α and the repression of PPARα. Moreover, GR knockout ECs have a defective metabolism with notably decreased FAO—a phenomenon previously linked with the profibrogenic phenotype. In this study, FAO was restored by inhibition of the Wnt pathway and fibrosis was restored by inhibition of PPARα agonist and fatty acid synthase. On the other hand, FAO inhibition worsened fibrosis and simvastatin treatment did not show a significant effect. Furthermore, GR loss increased expression of the pro-inflammatory cytokines IL-1β, IL-6, IL-10, IL-17, eotaxin or CCL4, and neutralisation of IL-6 by specific antibody also reduced fibrosis. At the end of incubation of tubular epithelial cells with culture media acquired from GR knockout HUVECs as well as endothelial and tubular epithelial cells carried from diabetic mice, there was a similar transition to profibrogenic and FAO-defective tubular ECs [78,79].

## 5. Role of Endothelial Glucocorticoid Receptor in the Pathogenesis of Chronic Kidney Disease

Chronic kidney disease is a global healthcare problem with high prevalence, multiple complications and increased mortality [80]. As chronic kidney disease advances, regardless of aetiology there is progressive fibrosis and kidney failure defined as a decline in GFR. Despite its significance, the pathophysiology of this process is not fully discovered and multifactorial. Some observatory studies link GFR decline with both endothelium dysfunction and chronic inflammation [81]. Furthermore, uremic toxins, particularly indolic compounds, accumulated due to worsening kidney function, have a direct negative effect on ECs [82]. On the other hand, common causes of kidney disease such as diabetes, hypertension and atherosclerosis strongly affect the endothelium [18].

As mentioned before, one of the scenarios leading to fibrosis in kidneys is endothelial to mesenchymal transition taking place in the glomeruli. In the case of kidney of fibrosis, a reduction in peritubular capillaries is considered as one of the key abnormalities. Animal studies of kidney fibrotic processes, regardless of aetiology, have shown major ultrastructural changes of the peritubular endothelium, mainly loss of fenestration and an increased number of caveolae and cytoplasmic vesicles accompanied with increased cell permeability [83]. Studies on mice have revealed the importance of peritubular endothelium and pericytes. Similarly to podocytes and the glomerular endothelium, those cells communicate with each other through VEGF and PDGFβ. In a model of interstitial kidney injury by both induced ischaemia and unilateral ureter obstruction, signalling from the endothelium to pericytes via PDGFβ or the other way round by VEGF, causes transition to fibrogenic cells and vasculature rarefaction. Those effects can be reversed by inhibition of VEGFR2 and PDGFR β. Furthermore, VEGFR stimulation causes the recruitment of inflammatory cells. Interestingly, those deleterious effects occur a few days after insult and are accompanied by a shift in VEGF isoform, mainly downregulation of VEGF164 [84]. Other authors have shown VEGF to be a factor protecting the peritubular endothelium from adverse ultrastructural alteration [83]. The nature of these conflicting results is still unclear and might be associated with the function of different VEGF isoforms. There is growing evidence of the importance of autophagy in kidney homeostasis and diseases. Autophagy is a mechanism of lysosomal degradation of cytosolic compounds which helps to maintain cell homeostasis and adaptation to injury. In kidneys, it is generally recognised as a protective process involved in cell regeneration after acute kidney injury. Animal and cellular research suggests that defective autophagy might contribute to diabetic kidney disease, focal segmental glomerulosclerosis (FSGS) and cyst growth in polycystic kidney disease [85]. Furthermore, studies on mouse cell lines have demonstrated that autophagy deficiency induces IL-6-dependent EndMT [86]. On the other hand, prolonged autophagy after kidney injury might be associated with interstitial fibrosis [85].

Current evidence demonstrates the importance of Wnt signalling in kidney disease. Under normal conditions, the Wnt pathway is active during embryogenesis and less profound in healthy kidneys. Nevertheless, in certain conditions such as polycystic kidney disease, diabetes or fibrosis it can be exerted and contribute to disease progression. While Wnt signalling is very complex and involves multiple ligands, generally it seems that its prolonged activation has a fibrotic and deleterious effect on multiple kidney cells such as tubular epithelial cells or podocytes [87,88]. In parallel to autophagy, Wnt signalling initially protects kidney cells after injury, promoting cell survival and regeneration, but leads to fibrosis if the process is prolonged [89]. Additionally, Wnt stimulation might lead to the reduction in klotho in kidneys and the cardiovascular system [90]. Moreover, there is growing evidence of a pathological effect of Wnt stimulation in cardiovascular disease. Numerous studies have shown a connection between the Wnt pathway and atherosclerosis, endothelial dysfunction, vascular calcification and heart failure. It is important to note that cardiovascular diseases are the leading cause of mortality in chronic kidney disease [91,92]. In a renal endothelium cell line, stimulation of the Wnt pathway through its ligand Dickkopf-3 is associated with EndMT and impaired angiogenesis [93]. There is growing evidence suggesting that GC treatment might suppress the canonical Wnt pathway through stimulation of its inhibitors or inhibition of its ligands [30,94]. On the other hand, Wnt inhibition might be partly responsible for some GC side effects such as osteoporosis or adipogenesis [95].

GCs also have a direct effect on heart contractility and myocardial hypoxia, playing an important role in the pathogenesis of heart failure. Mice with GR knockout in cardiomyocytes develop cardiac hypertrophy and left ventricle failure [14]. In addition, endothelial dysfunction measured as the hyperaemia peripheral arterial tonometry index is an independent risk factor of cardiovascular disease in patients with chronic kidney disease. Vascular calcification is one of the hallmarks of chronic kidney disease-associated cardiovascular disease [96]. While its aetiology is complex, studies show that GCs can initiate vascular calcification through apoptosis of vascular smooth muscle cells and osteoblastic differentiation. Novel data from in vitro studies demonstrate that GCs might also induce vascular calcification via MR signalling [97].

## 6. Role of Endothelial Glucocorticoid Receptor in Pharmacotherapy

For more than 50 years, GCs have been successfully used in a variety of diseases. Despite their efficacy in tackling numerous autoimmune, inflammatory or allergic diseases, chronic treatment can lead to many side effects, among them infections, myopathy, neuropsychiatric disorders and impaired metabolism [98]. Recently, a large cohort study of patients with common inflammatory risk showed a dose-dependent increase in cardiovascular disease hazard related to the use of GCs [99]. In addition, a mendelian study evaluating SERPINA6 genetic variants among a large population demonstrated the correlation of morning cortisol level with increased risk of ischaemic heart disease and heart failure. This effect has been related to the level of corticosteroid-binding globulin and the delivery of cortisol to peripheral tissues [100]. It is important to note that high doses of GCs can stimulate the expression of MR in HUVECs and that MR signalling contributes to vascular inflammation in mice [95,101,102]. In a randomised trial comparing the effects of methylprednisolone vs. placebo treatment in patients with IgA nephropathy, there was a significantly greater risk of serious infections in the GC group. Although the risk of end-stage kidney disease or a 40% decline in GFR after a 2.1-year median follow-up was decreased in the group treated with steroids, the trial has been terminated because of adverse events [103]. Similarly, another study comparing GC addition and intensive supportive therapy in patients with IgA nephropathy concluded that GC addition does not improve kidney function and increases the risk of adverse events—infections, weight gain and impaired glucose tolerance [104]. GCs can induce osteonecrosis. In vitro studies of human bone microvascular cells have demonstrated reduced expression of vasodilators and VEGF as well as induction of PAI-1 and ET-1 in reaction to GCs [105].

One of the major problems of GC therapy in kidney disease is GC resistance. The nature of this phenomenon is complex and not yet fully discovered and may differ among various cell types. Novel studies on EC lines show the importance of GR isoform balance, and post-translational and epigenetic modifications. One study on HUVECs explored the causative role of proteasome degradation in dexamethasone resistance. It proved that GC-resistant cell lines have higher expression of BAG-1—a proteasome-recruiting protein—increasing GR degradation [106]. Further studies revealed alternative GR promoter isoforms among dexamethasone-resistant and sensitive HUVECs. Resistant cells expressed more 1C and 1D isoforms but less 1F. Moreover, dexamethasone treatment further decreased the expression of 1C isoform and decreased GRα. This can be explained by the different methylation patterns in promoter isoforms. Dexamethasone-sensitive cells show higher 1D and lower 1F isoform methylation. Demethylation treatment diminished discrepancies in dexamethasone treatment in both HUVEC lines studied [107].

Those issues raised a need to lower the rate of adverse effects while maintaining clinical efficacy. One way is to deliver GCs directly into target cells, avoiding the exposure of tissues vulnerable to side effects. For example, researchers have been able to mitigate mouse anti-GBM glomerulonephritis by administering liposome-encapsulated dexamethasone conjugated with anti-E-selectin antibodies. This drug accumulates in inflamed kidney endothelium and causes the reduction in E-selectin, p-selectin and VCAM-1 expression. This action mitigates local inflammation [108]. A similar approach has been tested to locally deliver prednisone in rat renal ischaemia–reperfusion injury. This study demonstrated that liposomes are absorbed by macrophages both in cell cultures and in vivo in rats and can precisely deliver prednisolone to the place of inflammation [109]. Another method used macrophages incubated with dexamethasone to create dexamethasone-rich microvesicles. Those particles can deliver dexamethasone precisely to the inflamed kidney since they express integrins that can interact with VCAM-1 and ICAM-1 adhesion molecules on target cells, for example, glomerular ECs. Furthermore, microvesicles can carry additional GR particles. Microvesicle dexamethasone treatment in a murine model was shown to be superior to traditional dexamethasone treatment in the case of renoprotection and reduction in kidney inflammation in different models of kidney injury. The authors also demonstrated a reduction in adverse effects such as hyperglycaemia [110]. There are also ongoing studies evaluating the use of polymeric nanocarriers to selectively distribute GCs into kidney cells [111].

Secondarily, there are a number of studies investigating so-called selective GR agonists and modulators. Those compounds are capable of alternatively interacting with GR with preference to induce transrepression and not transactivation, which in theory should reduce side effects while maintaining clinical efficacy. Currently, due to very complex GR action, it does not bring the expected benefits but the clinical trials are still ongoing [112,113].

## 7. Conclusions

Glucocorticosteroids are multifunctional hormones that regulate a number of processes in the human organism. Recent studies have shown the role of endothelial glucocorticosteroid receptors in the regulation of renal function. It has also been shown that dysfunction of these receptors may be the cause of many renal diseases such as glomerulopathies, chronic kidney disease and diabetic kidney disease as well as arterial hypertension and vascular diseases. Alterations in the glucocorticosteroid receptors may affect the development and clinical course of these diseases, but may also affect the efficacy of glucocorticosteroid therapy. Knowledge of the precise role of glucocorticosteroid receptors in the pathogenesis of these diseases may contribute to a better understanding of their clinical course, better diagnostics and new therapeutic options. In addition, it has been shown that endothelial glucocorticosteroid receptors may be the therapeutic target in kidney diseases. Future research in this area should include the search for new selective modulators of glucocorticosteroid receptors that can be used in clinical practice, thus avoiding many of the adverse effects that accompany glucocorticosteroid therapy and may also reduce glucocorticosteroid resistance. However, a complete understanding of the role of endothelial glucocorticosteroid receptors in the pathogenesis and therapy of kidney disease requires further study.

## Figures and Tables

**Table 1:** Patomechanism and potential role of endothelial glucocorticoid receptor in the pathogenesis of particular diseases.

Endothelial Glucocorticoid Receptor in the Pathogenesis of Particular Diseases	Pathomechanism
Hypertension and vascular diseases	GCs increase AT1 receptor concentration and change the flow of Na^+^ and Ca^2+^ GCs can increase the secretion of ET-1 and AT-2 from ECs causing vascular smooth muscle contraction GCs directly reduce eNOS transcription
Cardio-vascular diseases	Wnt stimulation
Diabetic nephropathy	GCs affect eNOS and VEGF systems Wnt/β-catenin signalling impaired fatty acid oxidation (FAO) defective autophagy
Chronic kidney disease	Wnt signalling which leads to fibrosis GCs can initiate vascular calcification through apoptosis of vascular smooth muscle cells and osteoblastic differentiation

**Table 2:** Potential links between glomerulopathies and abnormal GR signalling.

Glomerulonephritis	Pathogenesis of Selected Glomerulonephritis
Focal segmental glomerulosclerosis (FSGS)	Defective autophagy
ANCA-associated vasculitis	NF-κB signalling in ECs mediated by ANCA-stimulated neutrophils
Minimal change disease	VEGF depletion
Lupus nephritis	Damage to ECs

## Data Availability

Not applicable.

## Abbreviations

ANCA	Anti-neutrophil cytoplasmic antibody
AP-1	Activator protein 1
AT1	Angiotensin II receptor type 1
AT-II	Angiotensin II
BAG-1	BAG family molecular chaperone regulator 1
bcl-2	B-cell lymphoma 2
BP	Blood pressure
CCL	CC chemokine ligand
COX-1	Cyclooxygenase 1
Dickkopf-3	Dickkopf-related protein 3
DUSP1	Dual specificity protein phosphatase 1
eNOS	Endothelial nitric oxide synthase
EC	Endothelial cell
EndMT	Endothelial to mesenchymal transition
ET-1	Endothelin 1
FKBP5	FK506 binding protein 5
FAO	Fatty acid oxidation
FPR1	Formyl peptide receptor 1
GATA	GATA transcription factor
GBM	Glomerular base membranę
GC	Glucocorticoids
GFR	Glomerular filtration rate
GR	Glucocorticoid receptor
GTP	Guanosine-5’-triphosphate
HDAC	Histone deacetylase
HIF1α	Hypoxia-inducible factor 1-alpha
Hsp	Heat shock protein
HUVEC	Human umbilical vein endothelial cell
ICAM-1	Intracellular adhesion molecule 1
IL	Interleukin
IL1R2	Interleukin 1 receptor, type II
iNOS	Inducible nitric oxide synthase
LDL	Low-density lipoprotein
LPS	Lipopolysaccharide
MAPK	Mitogen-activated protein kinase
MPK-1	MAPK *phosphatase*-*1*
MR	Mineralocorticoid receptor
mTORC1	Mammalian target of rapamycin complex 1
NCC	Sodium chloride cotransporter
NLRP	Nuclear binding oligomerization domain
NF-κB	Nuclear factor kappa B
NLPR3	NLR family pyrin domain containing 3
NO	Nitric oxide
PAI-1	Plasminogen activator inhibitor-1
PDGFβ	Platelet-derived growth factor beta
PPARα	Peroxisome proliferator-activated receptor alpha
ROS	Reactive oxygen species
SAP30	Sin3A-associated protein
SDF-1	Stromal cell-derived factor 1
siRNA	Small interfering RNA
Snail1	Zinc finger protein SNAI1
STAT3	Signal transducer and activator of transcription 3
sVEGFR	Soluble vascular endothelial growth factor receptor
TLR	Toll-like receptor
TNF-α	Tumour necrosis factor alpha
TNFSF10	Tumour necrosis factor ligand superfamily member 10
V2R	Vasopressin receptor 2
VCAM-1	vascular cell adhesion 1
VEGF	Vascular endothelial growth factor
VEGFR	Vascular endothelial growth factor receptor
Wnt	Wnt signalling pathway
